# Community structure of aquatic insects in the karstic Jadro River in Croatia

**DOI:** 10.1093/jis/14.1.54

**Published:** 2014-01-01

**Authors:** Biljana Rađa, Mate Šantić

**Affiliations:** 1 University of Split, Faculty of Science, Department of Biology, Teslina 12/III, 21000 Split, Croatia

**Keywords:** longitudinal gradient, multivariate analysis.

## Abstract

This study focused on the aquatic insect community in the longitudinal gradient and temporal scales of the Jadro River. The river was sampled for a period of ten years (2000– 2010), four times per year through the various seasons, along the river course. Sampling stations were selected in the upper, middle, and downstream parts of the river. A total of 21,852 specimens of aquatic insects belonging to six orders were obtained. The species determination confirmed 27 different species in the river. The data were analyzed by the multivariate methodologies of correspondence analysis and cluster analysis (unweighted pair group method with arithmetic mean) using the similarity index of Morosita for all ten years. Canonical correspondence analysis was applied to the data to check which of the mesured physicochemical variables significantly explained community variation. According to those data, significant variables for the upper station were water temperature and dissolved oxygen, and chlorides was the significant variable for the lower stations.

## Introduction

Observing the community structure of the aquatic organisms in streams and rivers is a very usefull tool for biological analysis of streams and rivers because those organisms have specific responses to changes in environment and physicochemical factors. Also, those changes are relatively easy to measure and interpret ( Karr and Chu 1999 ).

Karst is defined as a terrain, generally underlain by limestone or dolomite, in which the topography is chiefly formed by dissolving rock, and which is characterised by sinkholes, sinking streams, closed depressions, subterranean drainage, and caves ( Field 2002 ). A wide range of closed surface depressions, a welldeveloped underground drainage system, and strong interaction between the circulation of surface water and groundwater typify karst. Due to very high infiltration rates, especially in bare karst, overland and surface flow is rare in comparison with non-karst terrains (Bonacci 1987). From that point of view, karst rivers in Croatia represent unique habitats defined with specific physicochemical water parameters that influence the faunal composition of those rivers ( Rađa and Puljas 2010 ). The Jadro River is a typicall Mediterranean karstic river, as Munne and Pratt (2004) define in their work.

In Croatia, there is insufficient knowledge concerning insect community structures in rivers. Several authors have published concise data about aquatic insects in karstic rivers, but most of them are a part of benthic macroinvertebrates ( Habdija et al. 1997 ; Habdija et al. 2002 ; Habdija et al. 2003 ; Rađa and Puljas 2008 , 2010; Vučković et al. 2009 ). Belinić et al. (1993) and Ivković et al. (2007) disscussed the trophic importance of dipteran larvae and the ecological features of aquatic dance flies (Diptera: Empididae). Habdija et al. (2002) and Previšić and Popijač (2010) studied trichopteran larvae and the distribution of trichopterans along a karstic river. Popijač and Sivec (2009) listed the stoneflies in the area of the Plitvice Lakes and along the mediterranean river Cetina.

In an attempt to define the biotic index for the karstic rivers in Middle Dalmatia, we collected aquatic insects in a ten-year period (2000– 2010) through all four seasons and on different river substrate along the Jadro River. Our study was carried out to determine the distribution, abundance, and richness of species in the insect community structure of the karstic Jadro River, and to contribute to investigations of poorly known insect fauna of the Croatian karstic waters.

## Materials and Methods

### Study area

**Figure 1.**
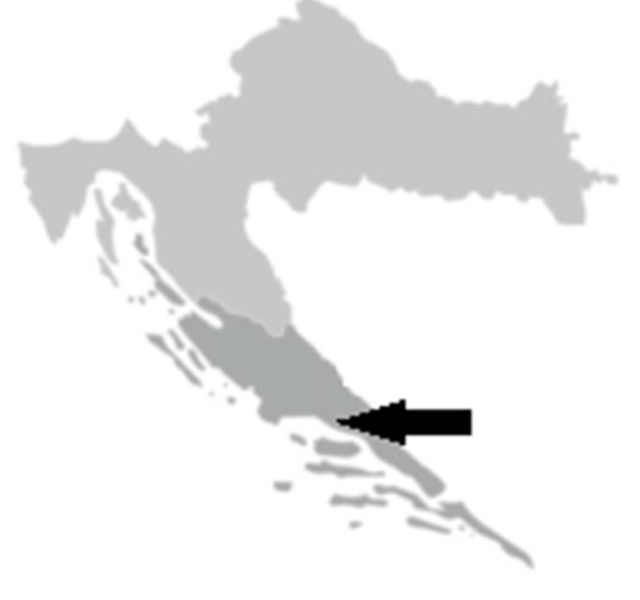
Karst area in Croatia. The Jadro River is marked by an arrow. High quality figures are available online.

The Dinaric karst occupies almost 50% of Croatian territory ( Figure 1 ). The investigated river, Jadro, is a small river originating from the underground waters of the Dinaric karst. The complete area consists of four paleo- dynamic and paleo-structural belts and belongs to carbonate rocks and karst phenomena along the Adriatic coast with carbonate forms from the Mesozoic and later ( Kuhta 2002 ). The Jadro River is a part of the Cetina catchment area together with the karstic rivers Ruda, Grab, and Žrnovnica ( Bonacci 1978 ). Our study is a part of a macroinvertebrate monitoring on a karstic river in Croatia according to the Water Frame Directive of the European Union.

**Figure 2.**
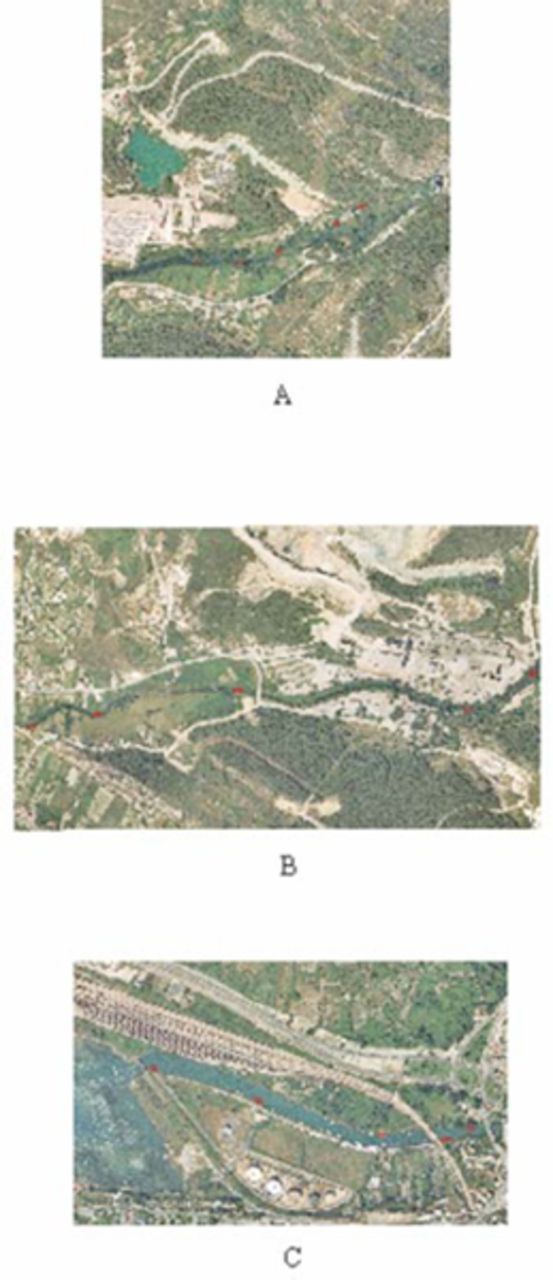
Watercourse of the Jadro River. (A) Upstream sites, (B) midstream sites, (C) downstream sites). High quality figures are available online.

Five sampling sites situated upstream ( Figure 2A ), midstream ( Figure 2B ), and downstream ( Figure 2C ) along the river course were examined. The upstream sites are directly influenced by groundwater, especially during the winter and spring seasons, while the midstream and downstream sites are influenced by agriculture and urban and industrial sources of pollution. The downstream sites of the Jadro River are influenced by seawater, especially during summer.

### Physicochemical parameters

Air and water temperature, dissolved oxygen, dissolved carbon-dioxide, alkalinity, hardness, pH, and dissolved salts were measured at each station four times per year from 2000 to 2010 (600 measurements total). Temperature, dissolved oxygen, dissolved carbon-dioxide, and pH were measured using a digital multimeter (Handylab set Schött, www.si-analytics.com ) with appropriate probes according to APHA (1995) . Surface water was sampled in 1-L polypropylene sampling bottles for alkalinity ( APHA 1995 ). The surface water hardness (in German degrees (d°H)) was calculated as the product of alkalinity (mg CaCO3/L) multiplied by a factor of 2.8. Substrate particle size analysis was determined using a modified Wentworth scale according to Bogner (1986) at the Institute of Oceanography and Fisheries in Split, Croatia.

### Insect sampling methods

One sample was taken at each station four times per year from 2000–2010 (total 600 samples) using a standard pond net (mesh size 500 µm). The fauna attached to stone surfaces were collected manually by tweezers and, if necessary, scraped with a fine brush or with entomological nets. The samples were placed in plastic bottles and preserved in 95% ethanol. They were sorted and identifiied to the lowest possible taxonomic level in the laboratory using a stereozoom Leica MZ 7.5 microscope (10x10 and 10x40, www.leicamicrosystems.com ). Genera was identified using available literature of the nearest region of Europe (Graf 1974; Hynes 1977 ; Chinery 1984 ; Dierl 1988 ; Campaioli et al. 1994 , 1999; Sansoni 1998 ; Harde 1999). Cluster analysis using presence/absence data with the Bray-Curtis similarity measure (for binary data it is equal to the Sørensen similarity index) and the group average clustering method were used to determine the faunistic similarity of insects between study sites. The graphics were made using Statistica 8.0 (StatSoft, www.statsoft.com ) and Primer 5.0 (Premier Biosoft, www.premierbiosoft.com ) software ( Clarke and Warwick 2001 ). The relationship between the insect taxa and the physicochemical parameters on the one side and sampling sites per year and season were analyzed by canonical correspondence analysis using XLSTAT 2010 software ( www.xlstat.com ). The complete data set was archived as a part of the Invertebrate Collection at the Department of Biology, Faculty of Science, The University of Split.

## Results

A total of 21,852 individuals of 27 different species were collected from the sampling sites along the water course. Twenty-seven genera from 20 different families were recorded ( Table 1 ). The number of specimens by species, location, and year are shown in Tables 4–6 . The locations with the highest diversity along the river course were middlestream sites during summer (32% of all recorded specimens). The lowest diversity was recorded at downstream sites during summer (only 2% of all recorded specimens). The highest taxa richness was observed among mayflies (order Ephemeroptera), with 10 different species represented. The abundance of insects increased in spring and summer (from 12% to 26%, respectively) and decreased in autumn and winter (6% and 8%). Stoneflies (Plecoptera) were not identified at midstream sites. On downstream sites, the most dominant order was Diptera, with families Chironomidae and Simulidae ( Table 1 ).

**Table 1:**
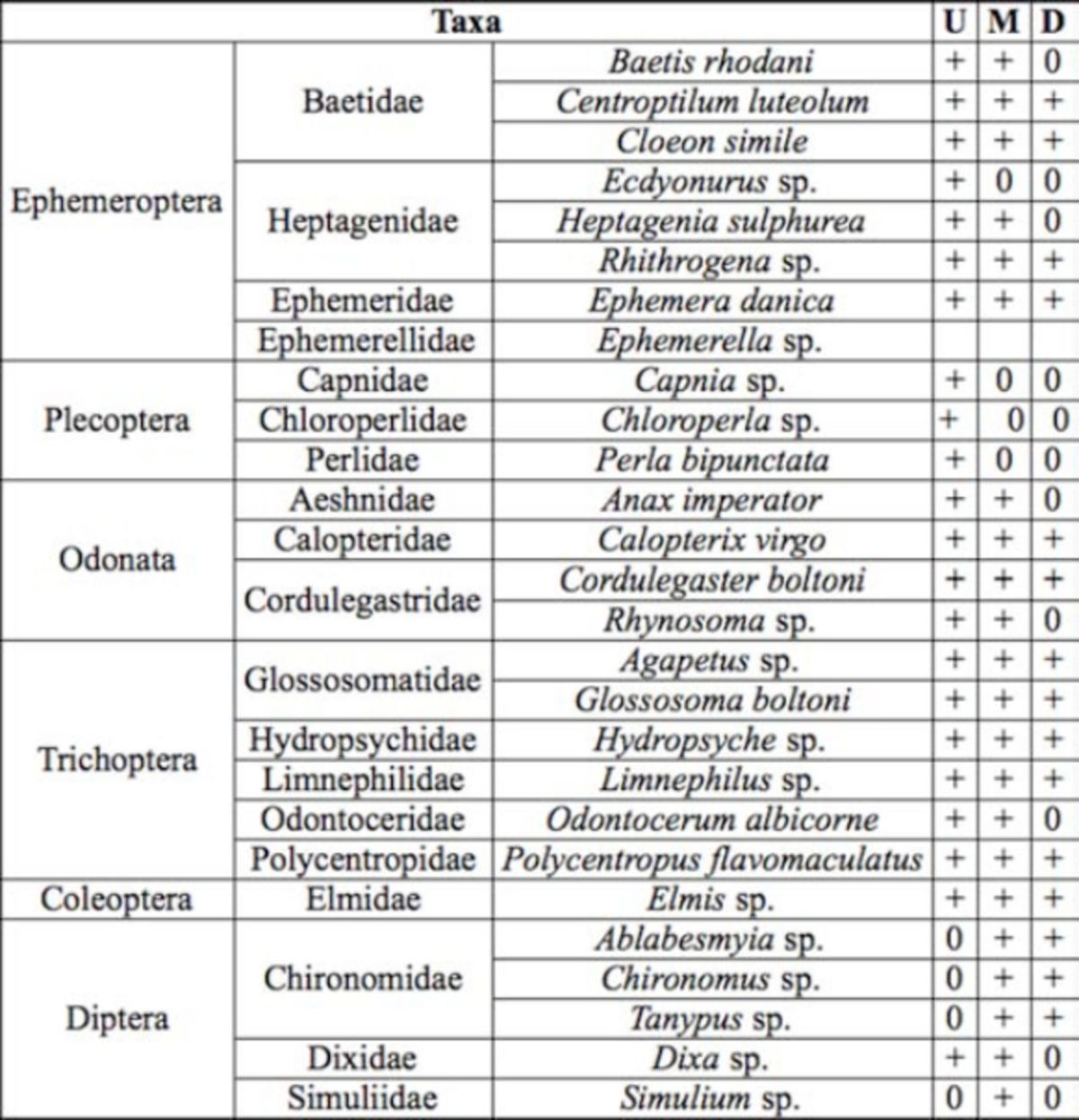
Composition of the benthic fauna of the karstic rivers. + - presence, 0 – absence, U-upstream sites; M-middstream sites; D- downstream sites.

**Table 4.**
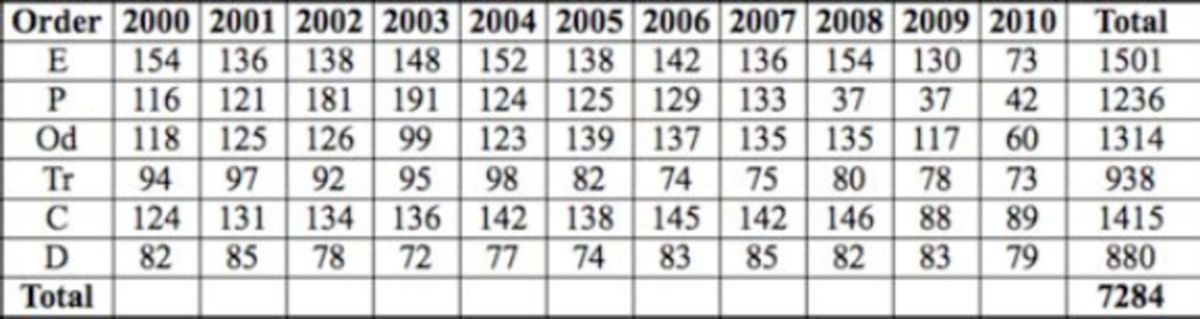
The number of specimens by year for upstream sites (E – Ephemeroptera, P – Plecoptera, Od – Odonata, Tr- Trichoptera, C – Coleoptera, D - Diptera).

**Table 5.**
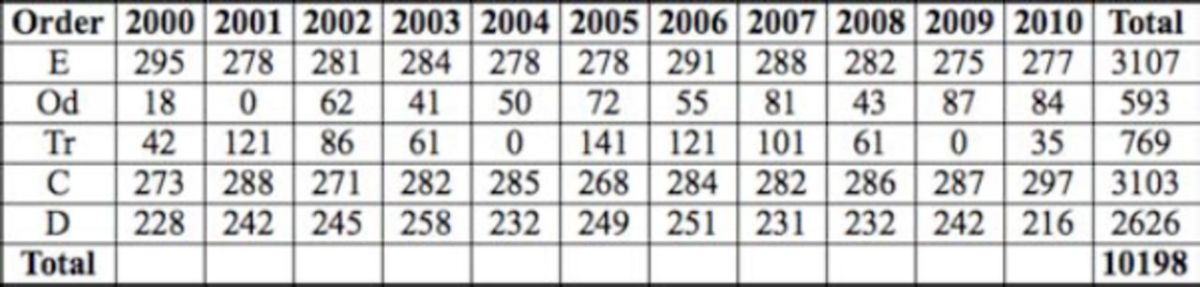
The number of specimens by year for midstream sites (E – Ephemeroptera, Od – Odonata, Tr- Trichoptera, C – Coleoptera, D - Diptera).

**Table 6.**
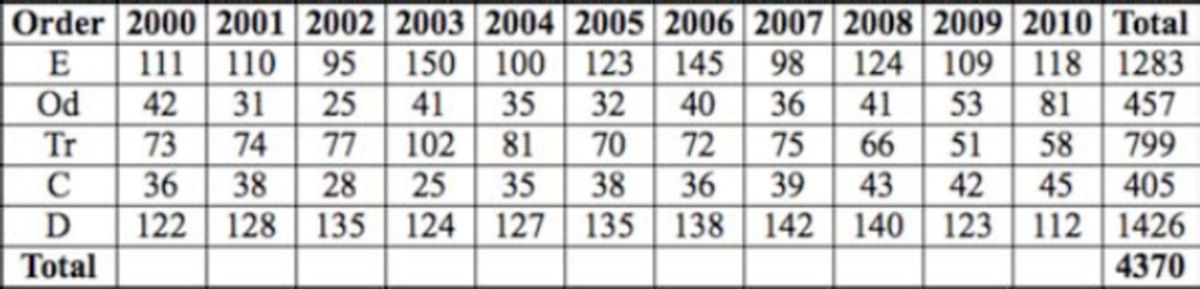
The number of specimens by year for downstream sites (E – Ephemeroptera, P – Plecoptera, Od – Odonata, Tr- Trichoptera, C – Coleoptera, D - Diptera).

**Figure 3.**
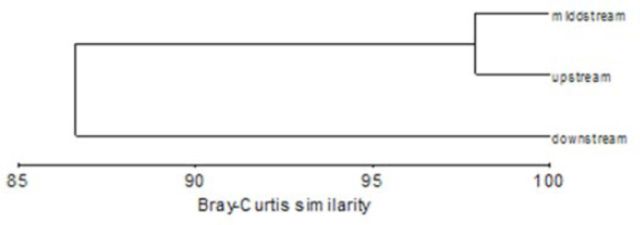
Dendrogram showing similarity between areas according to community structure and physicochemical parameters through seasons. High quality figures are available online.

The Sørensen’s index indicated similarity for upstream and midstream sites, between midstream and downstream sites, and between upstream and downstream sites ( Table 2 ). The unweighted pair group method with arithmetic mean (UPGMA) analysis confirmed that the downstream sites had the smallest similarity in comparison to the upstream and midstream sites, and the main factor for such distance was the sea influence on the downstream sites of the Jadro River. There was also a bigger dissimilarity between upstream and downstream sites than upstream and midstream sites because the influence of underground waters defined community structure at upstream sites as well as physicochemical parameters ( Figure 3 ).

**Table 2:**
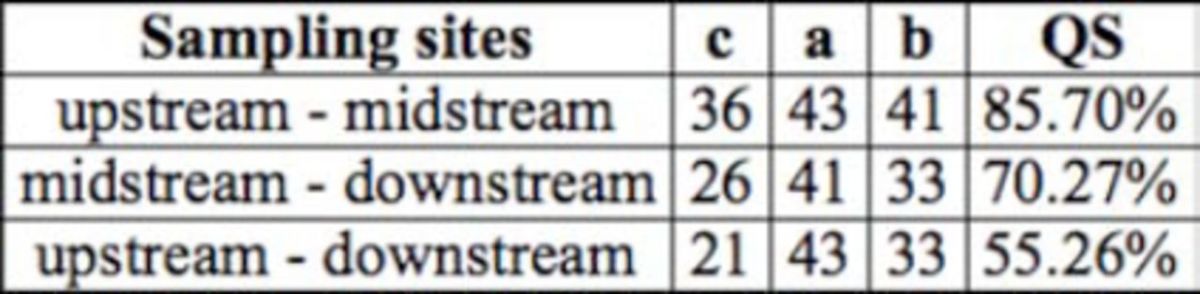
Sørensen’s index. QS – Sørensen’s index, c – number of commons species in two samples, atotal number of species in first sample, b- total number of species in second sample

**Figure 4.**
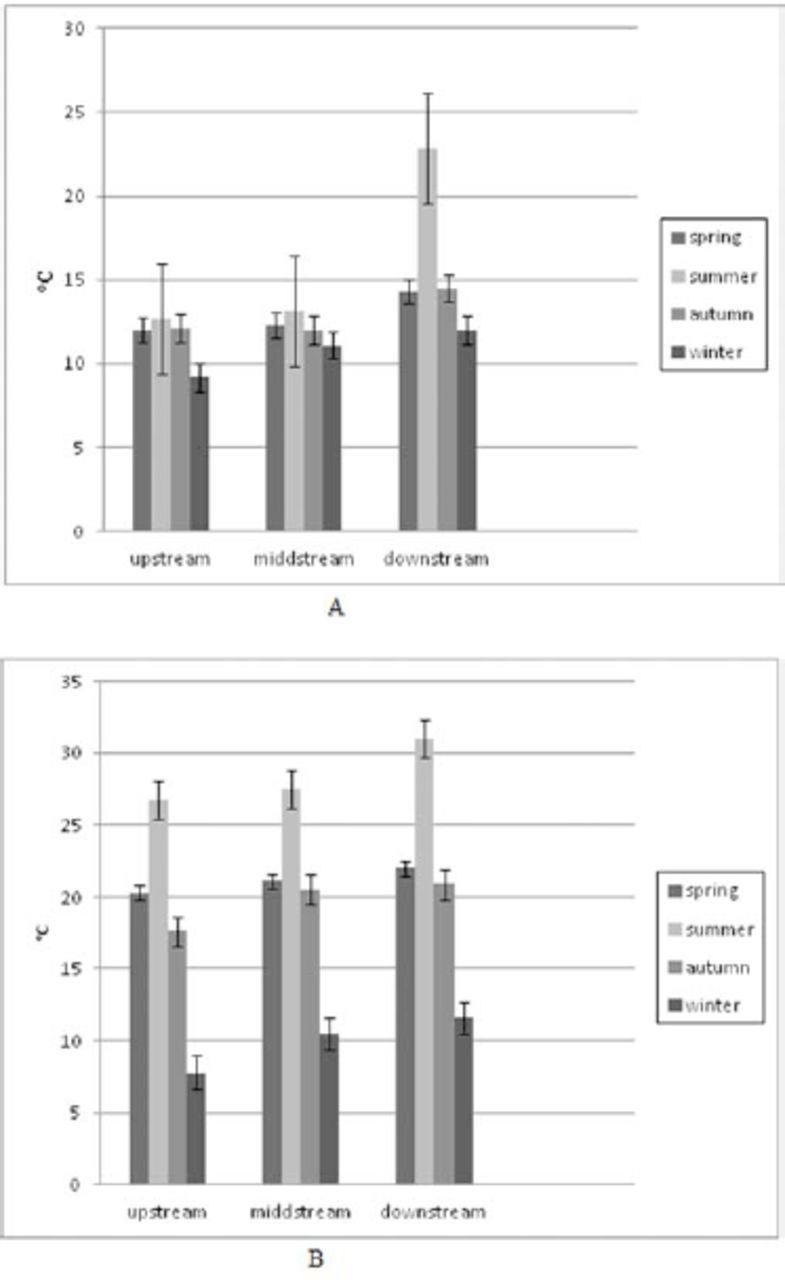
(A) Air temperature on sampling areas. (B). Water temperature on sampling areas. High quality figures are available online.

**Figure 5.**
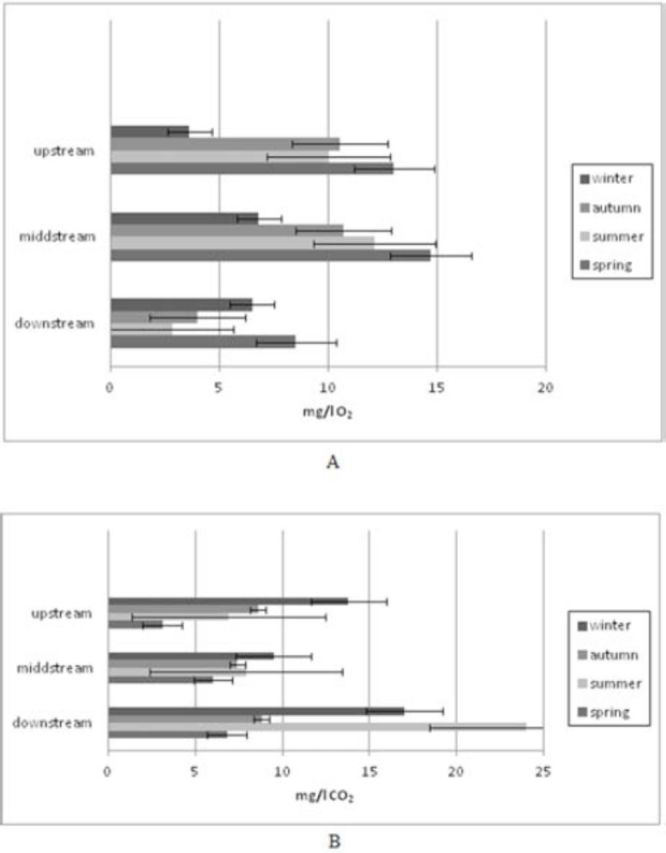
(A) Mean annual values of dissolved oxygen (mg/L). (B) Mean annual values of dissolved carbon dioxide (mg/L). High quality figures are available online.

**Figure 6.**
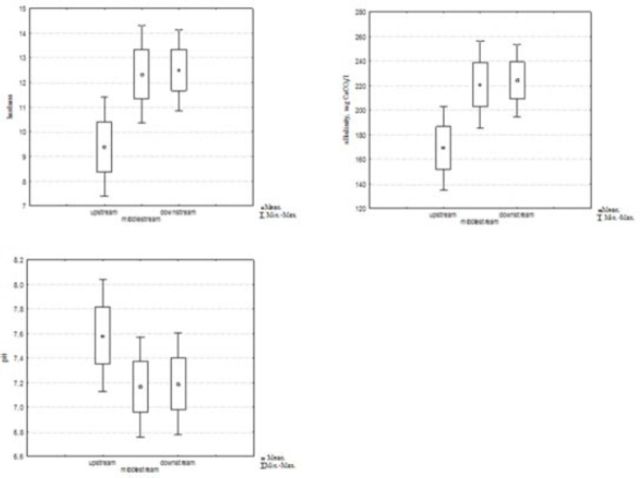
Hardness, alkalinity, and pH values. High quality figures are available online.

The granulometric analysis confirmed that the bottom substrate of upstream stations of all investigated rivers was characterized by coarse gravel material (4–64 mm grain size) and cobble (64–256 mm grain size), with substrate size declining downstream ( Table 3 ). The mean values of the water temperature varied from 9.2°C (upstream sites during the winter season) to 22.8°C (downstream sites during the summer season) following seasonal changes in the air temperature but with slow warming and cooling. The mean air temperatures showed the characteristics of dry Mediterranean climate, with values varying from 7.8°C (winter) to 31.0°C (summer). Spring and autumn values were mainly equal because there is no big difference between those two seasons in Mediterranean areas, including Middle Dalmatia ( Figure 4A , B). The mean values of dissolved oxygen at upstream sites varied from 3.6 mg/L (winter) to 13.0 mg/L (spring). The mean values at midstream sites varied from 6.8 mg/L (winter) to 14.7 mg/L (spring), whereas the downstream site dissolved oxygen values ranged from 2.8 mg/L (winter) to 8.5 mg/L (spring). A higher concentration of carbon dioxide was measured during the winter season at upstream (13.8 mg/L) and midstream sites (9.5 mg/L). At downstream sites on the Jadro River, the carbonate dioxide values were higher than those of dissolved oxygen in each season (6.8 mg/L– 24.0 mg/L). An extremely high value (24.0 mg/L) was recorded in summer (high water temperature, low flow, and velocity) ( Figure 5A , B). The hardness and alkalinity show the bicarbonate character of the water. The mean values varied from 150 mg CaCO3 /L (upstream sites) to 255 mg CaCO3/L (midstream and downstream sites). The pH value ranged from 6.8 to 7.8, with upstream sites having higher values ( Figure 6 ).

**Table 3.**
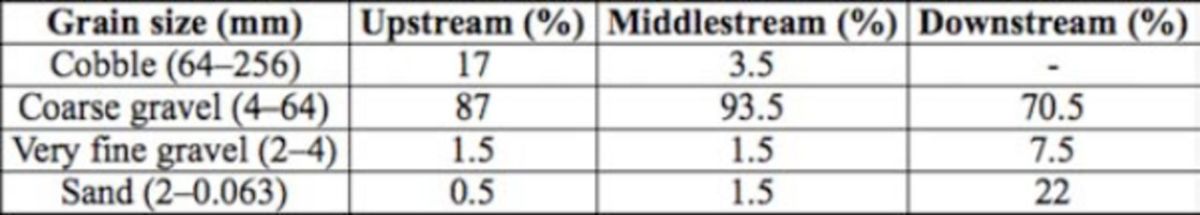
Granulometric analysis of sampling sites on investigated karstic river.

**Figure 7.**
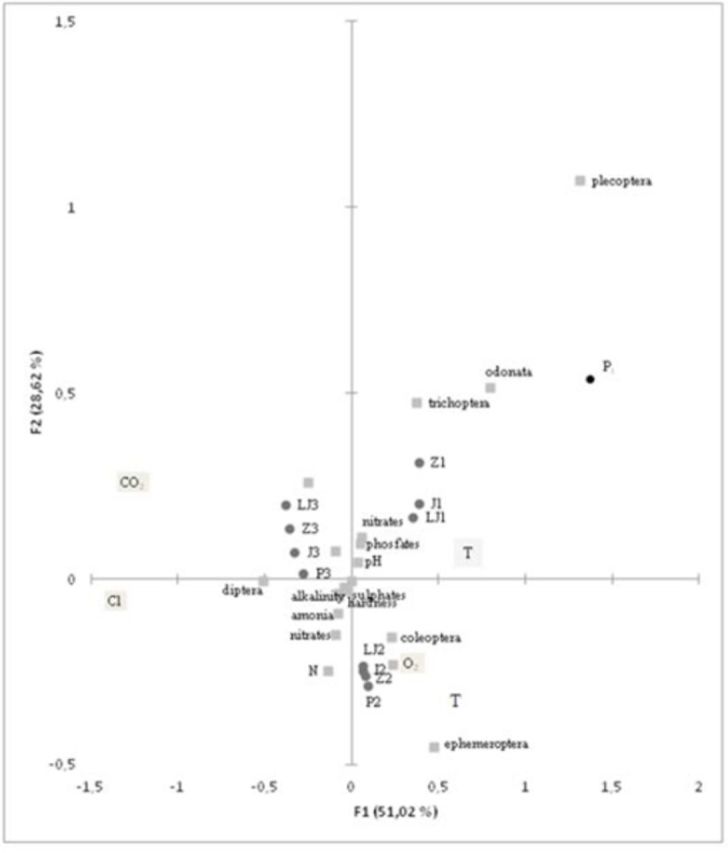
The ordination diagram of CCA analysis (1 – upstream sites, 2 – midstream sites, 3 – downstream sites; LJ – summer, J – autumn, Z – winter, P – spring). High quality figures are available online.

The CCA analysis ( Figure 7 ) showed that the first axes was most correlated with group Diptera. Their distribution was related to nitrogen, amonia, and sulphates from the agricultural areas as the main components that influenced the insect community at downstream sites (J3, Z3, and P3). The representatives of order Diptera were found on downstream sites during all seasons, showing a high concentration of dissolved carbon-dioxide and chlorides during summer (LJ3). This situation is a natural consequence of the antropogenic influence on water quality at the downstream part of the Jadro River. The concentration of chlorides is an indication of sea influence, especially during summer when the tidal regime is over the freshwater influence. The groups Trichoptera, Odonata, and Plecoptera are dependent on water temperature and on nitrates, phosphates, and pH values, which depend on underground flow and influence upstream sites during winter, autumn, and summer. Among them, Plecoptera were restricted to the upper part of the river and showed strong correlation with water temperature. The concentration of dissolved oxygen was in correlation with the distribution of Coleoptera and Ephemeroptera at middstream sites through the seasons. This part of the river showed the most stabile and consistent physicochemical values through seasons, so it can be considered the most favorable habitat for aquatic insects along the river course.

## Discussion

Mollusks, amphipods, and insects were the principal components of the community with regard to number of identified species, frequency of occurrence, and relative abundance in the karstic rivers in Croatia. This is a quite different community structure from that of the continental rivers in Croatia, in which chironomids and oligochaetes are the principal components ( Rađa and Puljas 2008 , 2010).

The macroinvertebrate community structure and abundance change seasonally and depend on the sampling site Fleituch (2003) . In Croatian karstic rivers, the most numerous group was snails (Gastropoda), followed by amphipods and insects. A considerable increase in the abundance of insects was observed in spring and summer, as supported by previous investigations ( Vuković 1981 ; Apostolska 1988 ; Kerovec 1996 ; Rađa 2002 , 2006).

Six groups of insects were recorded: mayflies (Ephemeroptera), stoneflies (Plecoptera), dragonflies (Odonata), caddisflies (Trichoptera), riffle beetles (Coleoptera), and true flies (Diptera). Mayflies (Ephemeroptera) were represented by eight different species. *Baëtis rhodani* (Pictet), *Heptagenia sulphurea* (Muller), and *Ecdyonurus* sp. were not isolated from the downstream samples. In earlier studies, 11 different species were identified, some of which are common in European freshwaters ( Di Giovanni et al. 2003 ; Rađa and Puljas 2008 , 2010). Stoneflies (Plecoptera) were isolated solely from the upstream samples because they develop only in cold, clear springs and are evidently sensitive to low oxygen concentration and organic pollution. Sivec and Popijač (2009) found adults of 15 stonefly species at Cetina River, but the stonefly fauna was not the same as found in the Jadro River. The main reason is the geographic location of the rivers. The Cetina River is situated in the continental part of the Dinaric karst, and the river course is mostly under the influence of the continental climate regime, while the Jadro River is under mediterranean climate regime. The physicochemical parameters that influenced insect community structure are quite different in those two freshwater habitats ( Štambuk-Giljanović 2005 ). Di Giovanni et al. (2003) identified the same species in the Chiascio River in Italy, as did Askew (1988) in his list of Odonata for Europe.

Odonata, Trichoptera, and Coleoptera were the most frequent at midstream parts of the Jadro River. The same distribution was recorded on the Cetina and Ruda rivers ( Vučković et al. 2009 ) as a part of the same catchment area. The authors indicated that abundance depends on the biomass of macrophytes and mosses.

Chironomides, as representatives of dipterans, were the most dominant insect group at the downstream part of the Jadro River. Their number increased during summer, when the dissolved carbon dioxide and water temperatures reach their maximum values. Chironomides are the most tolerant of negative human impacts ( Vučković et al. 2009 ), so their abundance in the sample is expected.

The Sørensen's similarity index confirmed great similarity between the upstream and midstream sites, lower similarity between midstream and downstream sites, and the lowest similarity between upstream and downstream sites. This proportion coincides also with the CCA analysis, so it can be presumed that the insect community of the investigated river is the result of specific physical and chemical conditions along the river course. The observed changes are more a result of the short term influence of the underground waters, climatic circumstances, and influence of the sea downstream. But today, the negative anthropogenic influence in the form of abiotic factors, such as the untreated reception of wastewater and flow regulation of the Jadro River, is obvious. So, the upstream part of the river is protected by The Law of Nature Conservation (NN 70/05, NN 139/08) as an ichthyological reservation.
